# Construction of crown profile prediction model of *Pinus yunnanensis* based on CNN-LSTM-attention method

**DOI:** 10.3389/fpls.2025.1567131

**Published:** 2025-07-09

**Authors:** Longfeng Deng, Jianming Wang, Jiting Yin, Yuling Chen, Baoguo Wu

**Affiliations:** ^1^ School of Mathematics and Computer Science, Dali University, Dali, China; ^2^ Dali Forestry and Grassland Science Research Institute, Dali, China; ^3^ Institute of Remote Sensing and Geographic Information System, School of Earth and Space Sciences, Peking University, Beijing, China; ^4^ School of Information Science and Technology, Beijing Forestry University, Beijing, China

**Keywords:** crown profile, convolutional neural network, long short-term memory, attention mechanism, crown profile competition index

## Abstract

*Pinus yunnanensis* is a significant tree species in southwest China, crucial for the ecological environment and forest resources. Accurate modeling of its crown profile is essential for forest management and ecological analysis. However, existing modeling approaches face limitations in capturing the crown’s spatial heterogeneity and vertical structure. This study aims to propose a novel approach that combines deep learning with a crown competition index to overcome the limitations of traditional models in capturing crown asymmetry and vertical structure, thereby enhancing prediction accuracy. Thus, we developed a hybrid CNN-LSTM-Attention deep learning model combined with a novel Crown Profile Competition Index (CPCI), based on data collected from 629 trees across five age-stratified permanent plots on Cangshan Mountain, Dali, Yunnan Province. Experimental results showed that the hybrid CNN-LSTM and CNN-LSTM-Attention models significantly outperformed the Vanilla LSTM model. In particular, the CNN-LSTM-Attention model achieved the best performance (MSE=0.00755 m^2^, RMSE=0.08691 m, MAE=0.05198 m, R²=0.98161), with absolute R² improvements of 0.16 and 0.17 over the Vanilla LSTM model by the CNN-LSTM and CNN-LSTM-Attention models, respectively. Additionally, the CNN-LSTM-Attention model demonstrated superior stability and performance in handling directional crown profile datasets. Incorporating CPCI improved prediction accuracy across all models, especially benefiting the Vanilla LSTM model. In conclusion, the proposed hybrid deep learning framework significantly enhances crown profile prediction for Pinus yunnanensis, and the introduction of CPCI provides a more precise representation of vertical and directional crown competition. This improvement facilitates more accurate assessment of tree crown dynamics, which is critical for understanding forest structure and competition.

## 1 Introduction

With the increasing demand for forest biomass and yield estimation, the importance of individual tree modeling has become increasingly prominent. The tree crown profile, as a key variable reflecting individual tree growth characteristics (Wang et al., 2022), not only represents the vitality and competitiveness of trees but also serves as a crucial indicator for assessing tree health and competitive status. The crown plays a vital role in processes such as photosynthesis, energy capture, and resource utilization (He et al., 2023; Jucker et al., 2025; Plaga et al., 2024), and directly influences various aspects such as forest dynamics simulations, stand productivity assessments, and biodiversity conservation (Meng et al., 2016; Harris et al., 2021). However, due to the time-consuming and labor-intensive nature of manual crown measurements, and the practical limitations of measuring the crown of every tree in field operations, there is an urgent need to develop high-precision crown profile models.

In recent years, significant progress has been made in the research on crown profile models, with various methods proposed to describe crown shape. Early studies approximated crown profiles using simple geometric shapes such as cones or ellipsoids (Rautiainen et al., 2008; Dong et al., 2016). While these methods were intuitive, they lacked flexibility and precision. Later, more sophisticated approaches, such as piecewise equations (Pretzsch et al., 2002; Sun et al., 2017), variable-exponent equations (Chmura et al., 2009; Crecente-Campo et al., 2013; Quan et al., 2020; Wang et al., 2019), and distribution functions (Ferrarese et al., 2015; Quan et al., 2020; Wang et al., 2019), were introduced to more accurately describe the morphological changes of crowns with height. With the diversification of methods for acquiring crown profile data, model fitting techniques have also advanced. For example, mixed-effect models, which combine fixed and random effects, effectively address hierarchical structure and heteroscedasticity issues (Hou and Chai, 2022; Zhou et al., 2023) and have been widely applied in modeling different species, such as *Pinus resinosa* and *Abies firma* in the Eastern United States (Dong et al., 2016). However, traditional statistical models, including mixed-effect models, typically rely on predefined functional forms and linear assumptions, which constrain their ability to capture highly nonlinear crown development patterns. They are also limited in modeling complex interactions among multiple influencing factors, especially when such interactions are dynamic and context-dependent.

Machine learning algorithms, with their capabilities for automatic feature extraction and the construction of nonlinear models, have been widely applied to crown profile modeling. Among these, Random Forest has been successfully utilized for predicting crown profiles across various tree species, outperforming traditional mathematical models (Tian et al., 2021). Additionally, ensemble methods such as Support Vector Regression (SVR) and Extreme Gradient Boosting (XGBoost) offer significant advantages in terms of prediction accuracy and robustness (Chen et al., 2022). However, these approaches rely on manual feature engineering and are prone to overfitting when handling high-dimensional data.

In response to these limitations, the advent of deep learning techniques has ushered in substantial breakthroughs in crown profile modeling. For instance, Gill and Biging (Gill and Biging, 2002) demonstrated the feasibility of simulating crown shapes using time-series models, laying the groundwork for more advanced methodologies. Building on their work, Long Short-Term Memory (LSTM) network has emerged as a powerful tool due to their ability to process spatiotemporal data effectively. The network can capture intricate patterns of crown profile changes with respect to both height and time, while also addressing common issues found in traditional methods, such as vanishing gradients and overfitting (Chen and Wang, 2023). Given these advancements, it is evident that integrating deep learning with other advanced algorithms holds great potential for developing more precise and reliable crown profile models. Therefore, future research should prioritize exploring these synergistic combinations to further enhance the accuracy and applicability of crown profile predictions.

Inter-tree competition and heterogeneous light environments are critical ecological factors influencing crown shape, directly resulting in asymmetric crown forms (Kong et al., 2021; Wang et al., 2023). In forest ecosystems, trees adjust the orientation and growth patterns of their crowns to adapt to surrounding environmental pressures, thereby optimizing resource acquisition. This adaptive growth behavior is particularly evident in aspects such as light capture, nutrient uptake, and spatial utilization. For instance, studies have shown that trees of different species can adjust the position and length of their crowns in response to competitive pressures, expanding toward directions with less competition or greater light availability to enhance light capture efficiency (Aakala et al., 2016; Krůček et al., 2019; Sun et al., 2022). These adaptive strategies, driven by competition and heterogeneous light environments, often result in significant directional differences in individual tree crown shapes. Given the impact of crown asymmetry on individual trees, it is crucial to account for these directional differences in modeling. Most crown profile models typically assume symmetrical crowns (Chen et al., 2022; Chen and Wang, 2023; Gao et al., 2021; Tong et al., 2024). However, this assumption overlooks the prevalent competition-induced asymmetries found in forests, thereby limiting the models’ applicability and predictive capabilities. Therefore, constructing directionally differentiated crown profile models is of particular importance.

*Pinus yunnanensis* is a major component of the coniferous forests in southwestern China, playing a crucial role in regional economic development and ecological restoration (Xu et al., 2016). However, research on its crown profile remains limited, particularly in artificial secondary forests. In addition, existing modeling approaches still present certain methodological limitations, especially regarding the accurate representation of spatial variation and directional differences in crown structure. To address such limitations in a more effective way, the main objectives of this study are as follows: (1) to develop a hybrid deep learning model that integrates CNN, LSTM, and a self-attention mechanism to improve the accuracy and adaptability of crown profile modeling; (2) to assess the stability of the proposed model by applying it to both non-directional and directional crown profile datasets, which reflect symmetrical and asymmetrical crown structures, respectively; (3) to explore the development of a crown competition index that can characterize competitive pressure across crown heights and directions, with the goal of improving the model’s effectiveness in crown profile prediction.

## 2 Materials and methods

### 2.1 Study area

**Figure 1:**
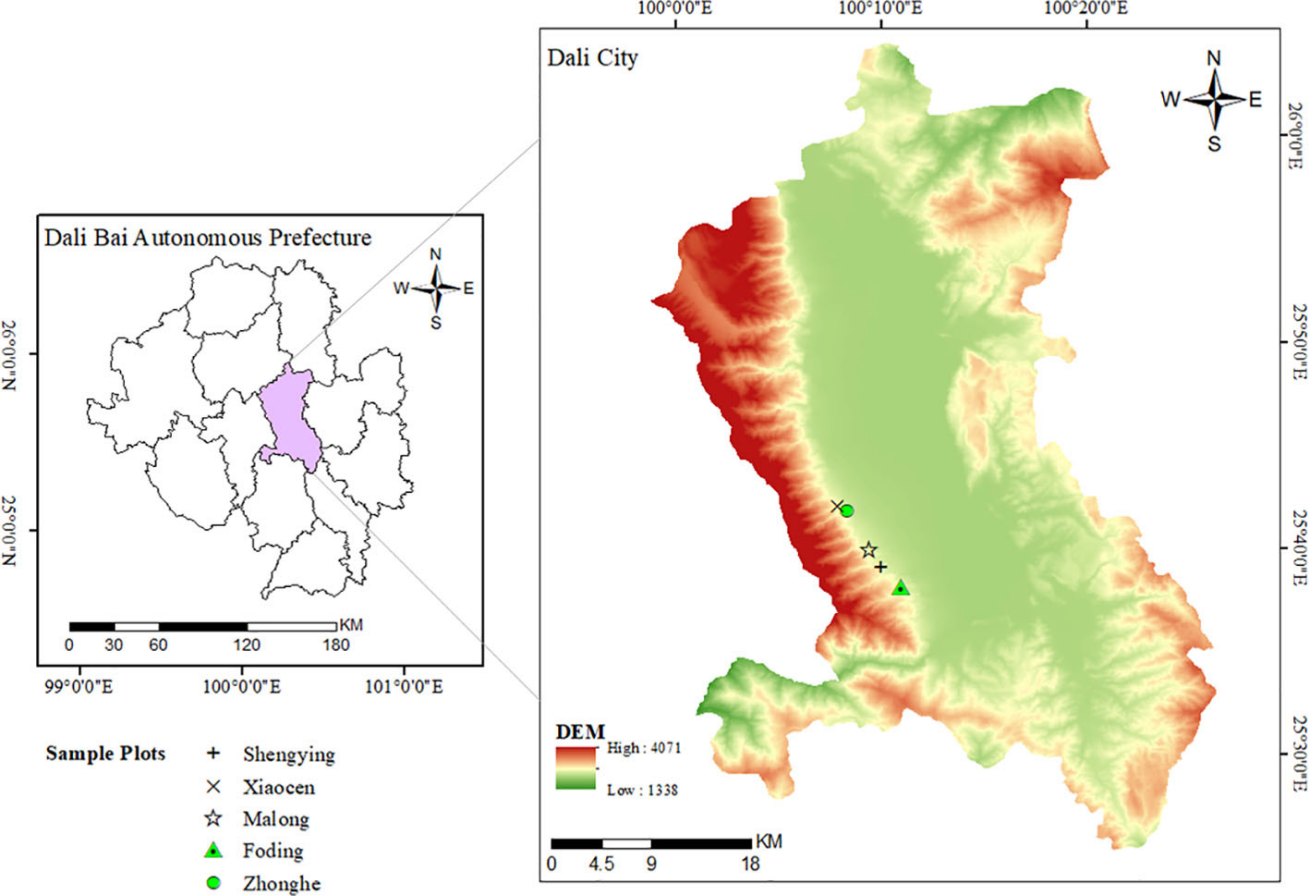
Location of the study area.

The investigation was conducted in the eastern sector of Cangshan Mountain, located within Dali City, Yunnan Province, China(25°34’~26°00’N, 99°55’~100°12’E). Elevations in the study area range from 1,966 meters to 4,122 meters above sea level, encompassing 19 distinct peaks within the Cangshan Mountain range. The highest point is Malong Peak at 4,122 meters, while the lowest elevation is found in the Dongpo Basin at 1,966 meters, resulting in a total elevation difference of 2,156 meters. Dali City is characterized by a typical subtropical highland monsoon climate, which features ample sunlight, substantial heat, slight annual temperature variations, pronounced diurnal temperature ranges, and clearly defined wet and dry seasons. The region maintains an average annual temperature of 16.1°C. Most precipitation occurs between May and October, culminating in an annual rainfall total of approximately 861.6 millimeters, with peak daily rainfall reaching up to 93.7 millimeters. The area experiences an average annual evaporation rate of 1,247.0 millimeters and maintains a relative humidity of 61%. Additionally, the region receives about 2,375.4 hours of sunshine each year. The predominant tree species in the study area include *Pinus yunnanensis*, *Pinus armandii*, *Betula alnoides*, *Vaccinium bracteatum*, *Ternstroemia gymnanthera*, and *Gaultheria griffithiana*. **Figure 1** illustrates the geographical boundaries and specific location of the research area.

### 2.2 Data collection and processing

**Figure 2:**
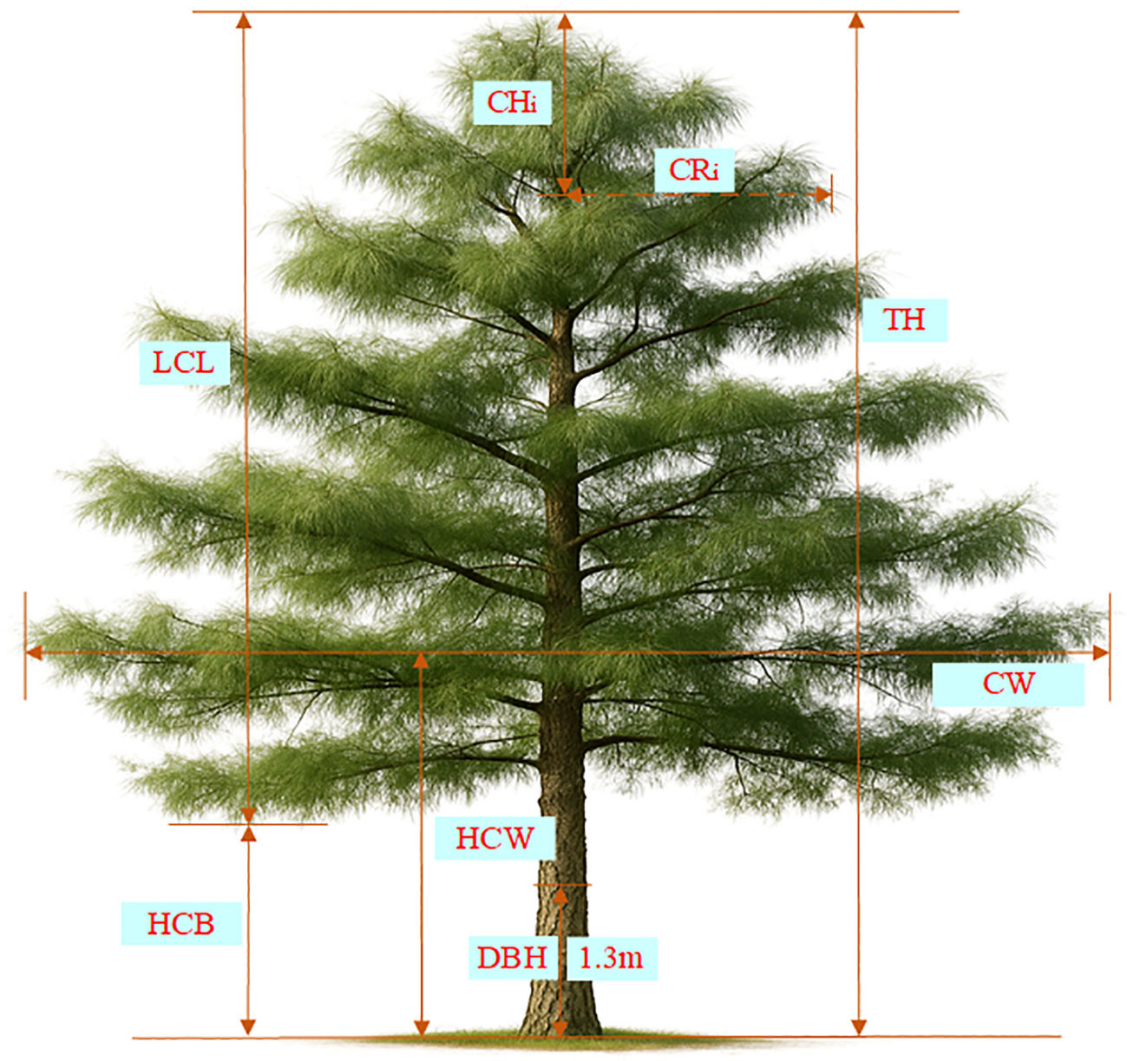
Tree Crown measurement diagram for *Pinus yunnanensis.* TH refers to total tree height; CH is the crown height from the treetop; DBH is the diameter at breast height (1.3 m); LCL represents the largest crown length; CR is the crown radius at CH; CW is the crown width; HCB is the height to crown base; HCW is the height at largest crown width.

The data for this study were collected from 5 circular survey plots on 5 peaks(Malong Peak, Shengying Peak, Xiaocen Peak, Zhonghe Peak and Foding Peak) of Cangshan Mountain, Dali City, Yunnan Province, China. **Table 1** shows the basic information for the various places. All trees with a diameter at breast height (DBH) of at least 5 cm were measured across five age-differentiated plots, with DBH recorded to the nearest 0.1 cm for each tree. In addition, the following measurements were taken using a ruler and a laser rangefinder: measurements were taken at four predefined relative crown heights (RCH=0.25, 0.5, 0.75, 1.0), representing specific proportional positions along the largest crown length (LCL), where the crown radius ( $CR_{i}$ ) was recorded in four cardinal directions (east, west, north, and south); total tree height (TH, m); and crown base height (HCB, m). Additional measurements included crown width (CW, m), crown height from the treetop to each measurement point ( $CH_{i}$ , m), the height corresponding to maximum crown width (HCW, m), and tree age, determined via increment core dating. All measurements were accurate to 0.1 m, except for DBH (0.1 cm) and tree age (recorded in years). The collected data were manually reviewed, and outliers and clearly erroneous values were identified and removed. Specifically, trees were excluded if any crown radius measurements were recorded at relative crown heights (RCH) exceeding 1.0, or if fewer than four valid crown radius values were available for each direction, to ensure consistency in the directional crown profile data. Finally, the dataset comprised 3,774 crown radius (CR) measurements from 629 trees aged between 10 and 52 years. **Table 2** provides detailed descriptions of all recorded variables. **Figure 2** shows a schematic diagram of the crown profile measurement variables.

**Table 1:** The basic information of each sample area.

Sample area	Elevation (m)	Slope (°)	Aspect	Plot radius (m, from center to edge)
Foding	2271	16.15	South	35
Malong	2195	17.7	Northeast	20
Xiaocen	2249	15.25	Southeast	35
Zhonghe	2254	12.85	East	35
Shengying	2284	30	Southeast	20

**Table 2:** Summary statistics of tree characteristic data for 629 sample trees.

Variable	Mean	Std Dev	Min	Max
DBH (cm)	19.5	6.8	5.4	42.1
TH (m)	11.8	3.1	4.3	22
CW (m)	2.1	0.7	0.5	5.3
HCB (m)	6.8	1.9	1.4	13.9
HCW (m)	7.7	2.2	0.9	13.5
CH (m)	2.9	2.4	0	16.4
CR (m)	1.0	0.7	0	5.1
LCL (m)	4.6	2.2	0.7	14.0
AGE (year)	27.3	7.1	10	52
CLR	0.4	0.1	0.1	0.8
TSC	0.6	0.2	0.3	1.7

Additionally, recognizing that the measured relative crown heights are not always equidistant, it was necessary to address this irregularity. During the interpolation process, several common methods were compared, including Lagrange interpolation, Newton interpolation, cubic spline interpolation, and Piecewise Cubic Hermite Interpolation Polynomial (PCHIP). While the first three methods can produce smooth curves, they often lead to overshooting, oscillations, or poor local control near data boundaries. In contrast, PCHIP preserves the shape and monotonicity of the original data, making it more suitable for crown profile interpolation. Therefore, PCHIP method was selected to interpolate the crown radius values at various crown height ( $CH_{i}$ ) measurement points. By interpolating four measured data points in each direction for each tree and including the data point with a crown radius of 0 at the top of the tree, eleven interpolated data points were obtained. These interpolated points, designated as $CR_{0}$ to $CR_{10}$ , represent the crown radius at 11 relative crown heights ranging from the crown top to the base. The relative crown heights are uniformly spaced at intervals of 0.1 (from 0 to 1), ensuring consistent vertical positioning within the crown structure.

**Figure 3:**
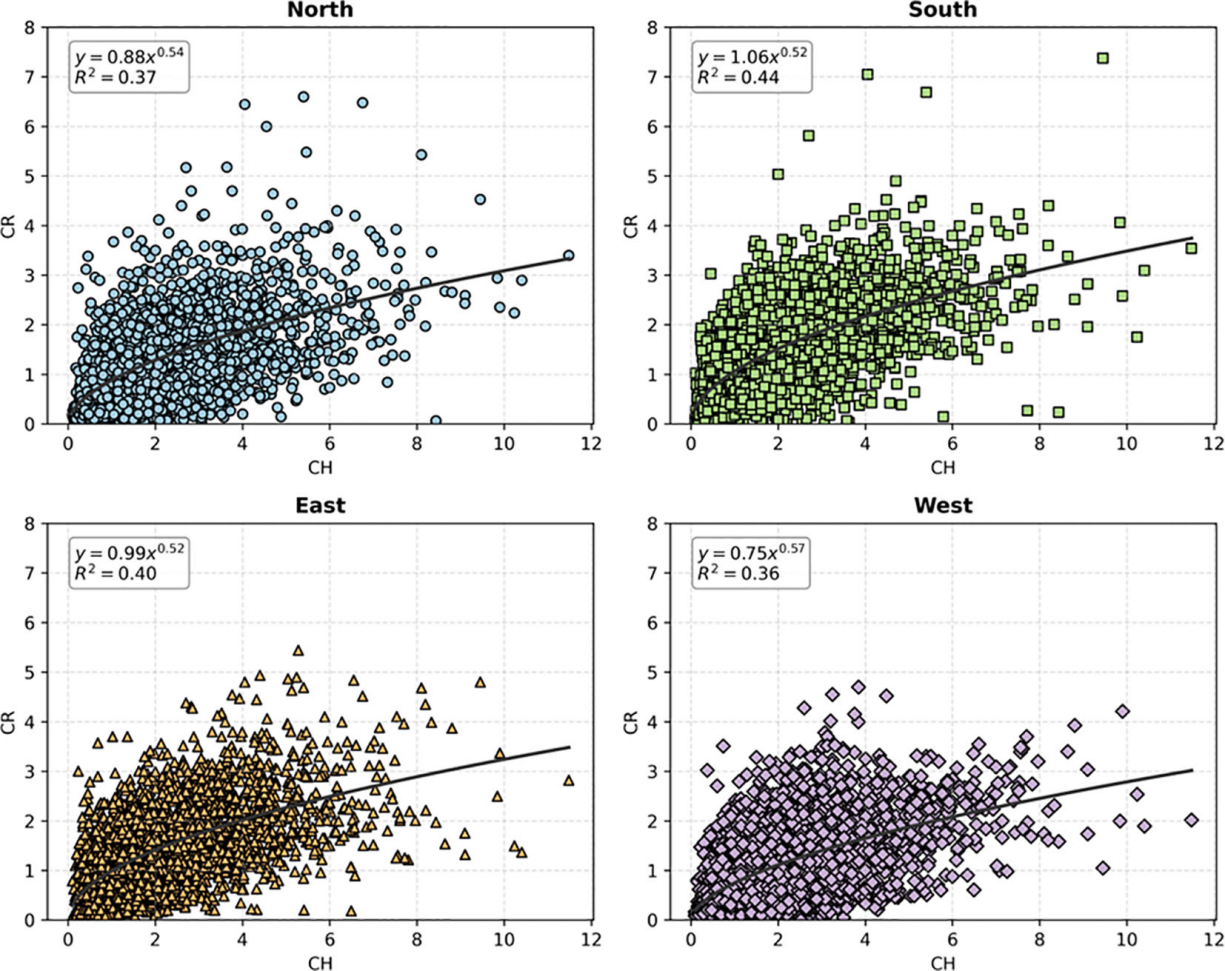
CR and CH scatter plots with power function regression in four directions.

Through scatter analysis of tree crown radius data in different directions (**Figure 3**), it can be seen that there are significant differences in crown profiles across directions. In order to describe its growth characteristics and spatial structure more accurately, it is necessary and valuable to make directional prediction of crown profile.

### 2.3 Crown competition index

#### 2.3.1 Determination of competing tree

In the study of forest competition, the determination of competing trees is the core task. The traditional methods mainly include fixed semi-circle method, fixed number of trees method and fixed region method, but these methods have obvious defects. For example, the radius scale of the fixed semi-circle method is not uniform (Hegyi, 1974; Holmes and Reed, 1991; Biging and Dobbertin, 1995), while the fixed number of trees method faces controversies regarding the selection of tree numbers (Hui et al., 2013). Although selecting four neighboring trees can achieve over 80% of the effectiveness of selecting eight neighbors, there is no standardized criterion for this selection process. Consequently, these traditional methods often result in either the over-selection or omission of competing trees. In response to the above problems, some scholars (Liu et al., 2023; Huang et al., 2024) proposed to use Voronoi diagram to analyze the spatial structure of stand, and then determine the number of adjacent trees to identify competing trees. Voronoi diagram is based on the coordinate of the target tree to construct its spatial competition structure unit, and the adjacent trees are uniquely determined by the common sides of Voronoi polygon. In this study, Voronoi diagram was used to determine the competing units, and then the competing trees of each subject tree were determined.

#### 2.3.2 Calculation of crown overlap area of different crown layers

After the unique determination of competing trees by Voronoi diagram, the tree crowns at different heights can be simplified into a circle, and the tree crowns at different heights can be calculated by taking the trunk of the subject tree as the center. Based on previous studies (Lian et al., 2024) and practical field experience, a vertical height difference ( ΔCH ) of 2 meters or less between the k -th layer of the subject tree and the t -th layer of the competing tree was used as the threshold to determine crown overlap competition. The specific formula is shown below.


(1) $$\begin{array}{c} \Delta CH=\left|CH_{i}^{k}-CH_{j}^{t}\right|\leq 2 \end{array}$$

Where, $CH_{i}^{k}$ represents the crown radius height of the k layer of subject tree i ; $CH_{j}^{t}$ represents the height of the crown radius of the t layer of competing tree j ; ΔCH represents the relative difference between the height of the k layer crown radius of the subject tree i and the height of the t layer crown radius of competing tree j .

**Figure 4:**
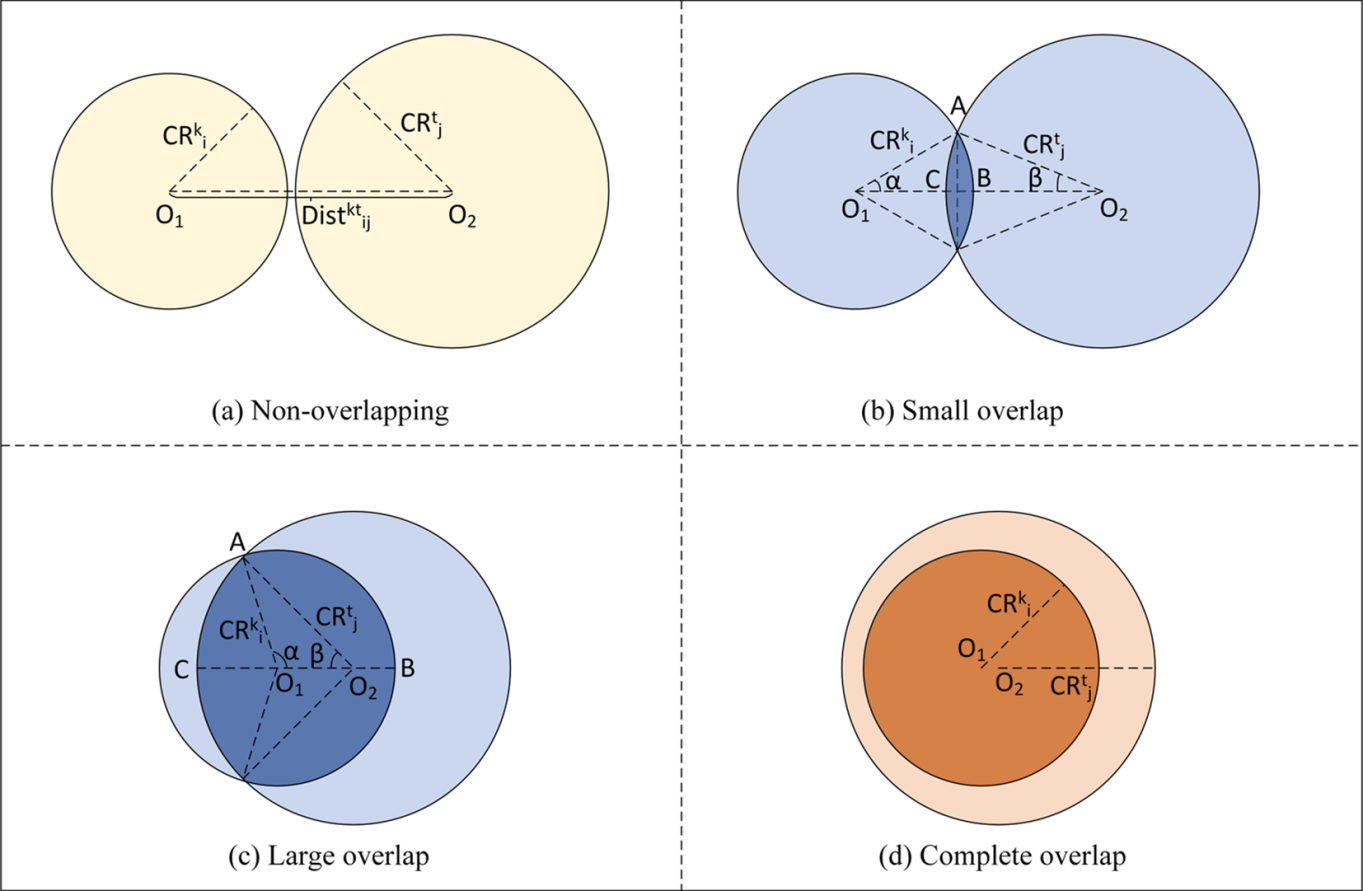
Diagram of tree crown overlap illustrating four overlap types: **(a)** Non-overlapping, **(b)** Small Overlap, **(c)** Large Overlap, and **(d)** Complete Overlap.

Crown overlap area is determined according to the distance between the subject tree and the competing tree and the relationship between the tree crown radius of the two trees, which can be divided into the following four cases (**Figure 4**):

In **Figure 4a**, $Dist_{ij}^{kt}\geq CR_{i}^{k}+CR_{j}^{t}$ indicates that the crown of the subject tree at the k layer height is separate from the crown of the competing tree at the t layer height, the overlapping area is 0, and there is no competition relationship. The calculation formula of the tree crown overlap area is shown in Equation 2:


(2) $$\begin{array}{c} AO_{ij}^{\left(k,t\right)}=0 \end{array}$$

Where, $AO_{ij}^{\left(k,t\right)}$ represents the overlapping area between the k layer height crown of the i -th subject tree and the t layer height crown of the j -th competing tree.

As shown in **Figures 4b, c**, when the crown of the subject tree overlaps but not completely overlaps with that of the competing tree, that is, when $\left|CR_{i}^{k}-CR_{j}^{t}\right|\leq Dist_{ij}^{kt}\leq CR_{i}^{k}+CR_{j}^{t}$ and the height difference condition set in this study is met, it is believed that there is a competitive relationship between the two. The calculation formula of the tree crown overlap area is shown in Equation 3:


(3) $$\begin{array}{l} AO_{ij}^{\left(k,t\right)}=\arccos\left(\frac{CR_{i}^{k^{2}}+Dist_{ij}^{kt^{2}}-CR_{j}^{t^{2}}}{2\times Dist_{ij}^{kt}\times CR_{i}^{k}}\right)\times CR_{i}^{k^{2}}\\ +\arccos\left(\frac{CR_{j}^{t^{2}}+Dist_{ij}^{kt^{2}}-CR_{i}^{k^{2}}}{2\times Dist_{ij}^{kt}\times CR_{j}^{t}}\right)\times CR_{j}^{t^{2}}\\ -2\times \sqrt{p\left(p-CR_{i}^{k}\right)\left(p-CR_{j}^{t}\right)\left(p-Dist_{ij}^{kt}\right)} \end{array}$$

Where, $CR_{i}^{k}$ represents the k layer height crown radius of subject tree i , and $CR_{j}^{t}$ represents the t layer height crown radius of adjacent tree j . $Dist_{ij}^{kt}$ represents the distance between the trunk of subject tree i and competing tree j , and p is Helen’s formula, which is the triangle area calculation formula.

As can be seen from **Figure 4d**, when the k layer crown of the subject tree completely overlaps with that of the t layer crown of the competing tree, that is, when $Dist_{ij}^{kt}\leq \left|CR_{i}^{k}-CR_{j}^{t}\right|$ , and the height difference condition set in this study (Equation 1) is satisfied, it is considered that there is a competitive relationship between the two trees. In this case, the crown overlap area is discussed. When the crown radius of the subject tree is greater than or equal to the crown radius of the competing tree, the crown overlap area is the crown area of the competing tree. When the crown radius of the subject tree is smaller than that of a competing tree, the overlapping area of its crown is the crown area of the subject tree. See Equation 4 for the specific calculation formula:


(4) $$\begin{array}{c} AO_{ij}^{\left(k,t\right)}=\{\begin{array}{c} \pi \times CR_{j}^{t^{2}},CR_{i}^{k}\geq CR_{j}^{t}\\ \pi \times CR_{i}^{k^{2}},CR_{i}^{k}<CR_{j}^{t} \end{array} \end{array}$$

Based on the above formula for calculating the crown overlap area of the subject tree and a single competing tree, the total crown overlap area of the subject tree and all competing trees at different height levels can be obtained (Equation 5):


(5) $$\begin{array}{c} AO_{i}^{\left(k\right)}=\sum_{j=1}^{N}\sum_{t=1}^{M}AO_{ij}^{\left(k,t\right)} \end{array}$$

Where, k represents the relative height of the subject tree itself at the radius of the crown of the subject tree; N indicates the number of competing trees. M represents the number of interpolated relative crown height levels (excluding RCH=0, where the crown radius is defined as zero). Accordingly, M equals 10, based on 11 total levels ranging from RCH=0 to 1.

#### 2.3.3 Crown profile competition index

The Crown Competition Index (CI) is an important index used to assess the intensity of competition between trees. The calculation of CI is usually based on the size of the tree crown and the relative position between them, reflecting the degree to which trees compete within the same space. In the process of tree growth, the expansion of the crown is not only affected by horizontal competition, but also the allocation and competition of resources in the vertical direction. In order to study the competition degree of tree crown radius at different heights, based on the crown overlap area at different heights, we referred to the Bella Competition Index (Bella, 1971) and the competition index based on crown overlap area proposed by Wang (Wang, 2017). On this basis, we developed a Crown Profile Competition Index (CPCI) to explore how the crown radius at various height levels is influenced by competition from neighboring trees. By calculating the CPCI of each height level, the spatial competition of trees at different growth heights can be understood in more detail, especially when simulating how trees cope with competitive pressures in complex environments, thus providing a more accurate model for predicting the crown radius of different height levels. The crown competition index CPCI of different height levels of the subject tree is shown in Equation 6:


(6) $$\begin{array}{c} CPCI^{k}=\frac{1}{Z_{i}^{k}}\times AO_{i}^{\left(k\right)}\times \frac{CH_{j}^{t}\times CR_{j}^{t}}{CH_{i}^{k}\times CR_{i}^{k}} \end{array}$$

Where, $CPCI^{k}$ represents the crown competition index of the k layer height of the subject tree; $AO_{i}^{\left(k\right)}$ represents the total crown overlap area between the crown height of the k layer of subject tree i and all competing trees; $Z_{i}^{k}$ represents the projection area of the crown of subject tree i at the height of the k layer.

At the same time, in order to reduce the computational complexity of data, traditional methods averaged tree crown radii in four or more directions to obtain the average crown radius of a certain height, while the distribution of tree crown radii in all directions has a certain degree of spatial heterogeneity (J. Wang et al., 2023). Tree crown width also competes in different directions, and this competition may be affected by environmental factors such as slope direction, light, soil, etc (Sun et al., 2022; Tian et al., 2024). Therefore, in order to achieve a more detailed directional crown profile model, this study proposed a CPCI allocation formula based on height level and direction (Equation 7). Assigning the crown competition index to different directions helps to more accurately describe spatial competition among trees, especially in densely forested areas or areas where competition effects are evident. In this way, the competition effect will be adjusted according to the actual orientation of the tree, which is more in line with the actual situation of tree growth and competition in nature, and can more accurately capture the growth of the tree in all directions, thus improving the accuracy of the model prediction.


(7) $$\begin{array}{c} CPCI_{Direction}^{\left(k\right)}=CPCI^{\left(k\right)}\times \left(1-\left(CR_{Direction}^{k}/\sum_{Direction}CR_{Direction}^{k}\right)\right) \end{array}$$

Where, $CPCI^{\left(k\right)}$ represents the crown competition index of the k layer height of the subject tree; $CR_{Direction}^{k}$ indicates the radius of the crown in a direction of the k layer height of the subject tree; Direction represents the crown direction of the subject tree (the crown radius direction of this study has four directions: east, west, north and south); k stands for the relative height of the subject itself at the radius of the crown of the subject.

### 2.4 Construction of crown profile model

#### 2.4.1 Feature selection and processing

Relevant literature (Tian et al., 2021; Chen et al., 2022) indicates that CR is primarily affected by AGE, DBH, TH, CW, HCW, HCB, CH, LCL, TSC, CLR and other variables. Additionally, since the data originate from different plots, we also incorporate basal area (BA) and stand density index (SDI) alongside these features. Moreover, CR is affected not only by the surrounding environment, but also by the upper spatial structure of CR. For example, Ventre-Lespiaucq et al. (2018) found that the upper crown can achieve light interception of the lower crown through its structural characteristics (leaf aggregation degree and crown openness), thus forming competition with the lower crown. Therefore, in this study, the CR data of tree crown radius at a certain interval is used as new feature information, so that the model can fully learn the spatial features of the data. CPCI is a competition index that quantifies the competition intensity based on the calculation of the crown radius of different crown layers and the crown overlap area of competing trees on the vertical spatial structure of a single tree scale in each plot. In this study, the competition index and the above characteristics together constitute the prediction of the crown radius of different crown layers.

Finally, the modeled data has 14 input variables and 1 output variable. The 14 input variables are: AGE, DBH, TH, CW, HCW, HCB, CH, LCL, TSC, CLR, BA, SDI, CR_(i-1)_, CPCI.

In the construction of crown profile model, different variables have different dimensions and value ranges. These variables of different dimensions may cause the model to pay too much attention to the variables with larger values and ignore the variables with smaller values during the training process, thus affecting the prediction accuracy of the model. In order to unify the influence of index values on the model, the data normalization method was adopted in this study to carry out dimensionless sample data to the same numerical range ([0,1]) to ensure that each input variable received equal attention in the model, thus improving the training effect and prediction accuracy of the crown profile model.

#### 2.4.2 CNN-LSTM-attention model

To predict crown radius across different heights, we developed a hybrid CNN-LSTM model as the core framework for crown profile prediction. In this structure, CNN is used to extract local spatial features, while LSTM captures dependencies between crown height layers by treating the crown profile as a pseudo–time series. Building upon this CNN-LSTM architecture, we further incorporated an attention mechanism to construct a CNN-LSTM-Attention model, aiming to enhance the model’s ability to focus on critical information at different height levels.

**Figure 5:**
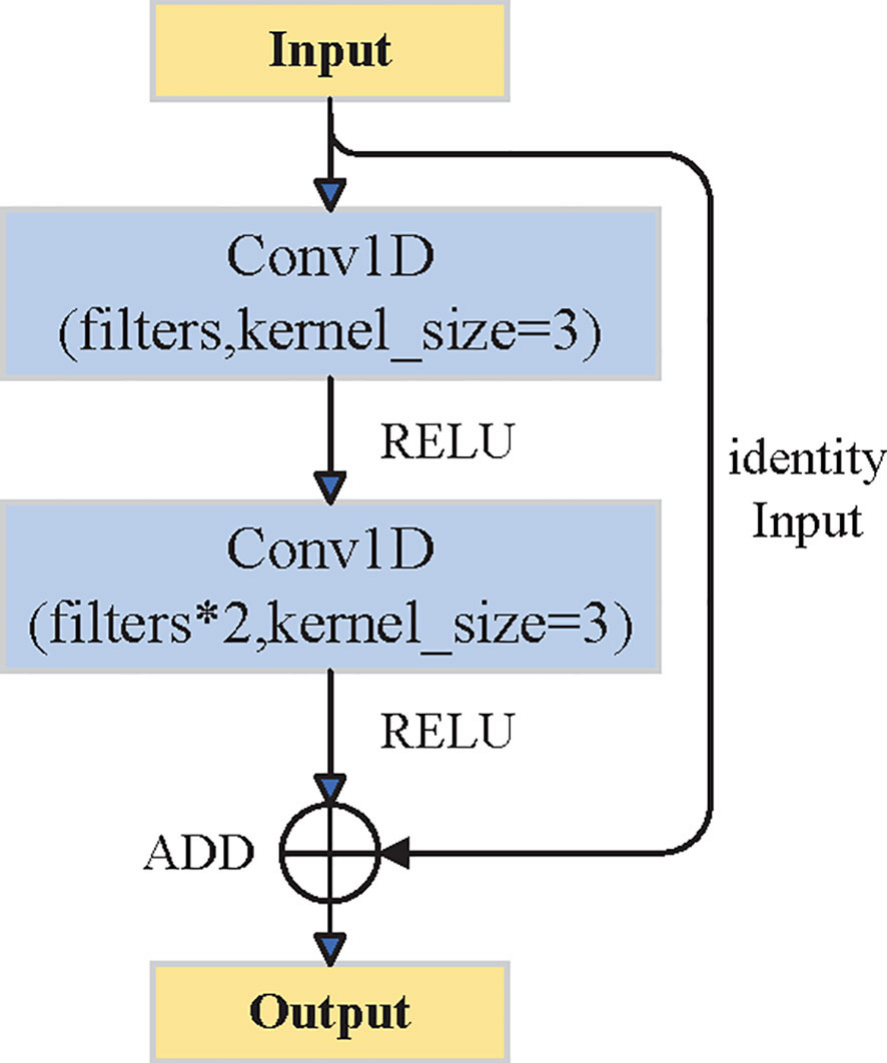
CNN structure.

In this study, the CNN structure was modified based on the residual network (ResNet) to model crown profiles more effectively. It extracts useful features from input variables while reducing the influence of irrelevant information, thus improving crown radius prediction. As shown in **Figure 5**, the CNN includes convolutional layers and residual connections. The input data were normalized to ensure consistent scale. Then, the normalized feature sequence was passed through two one-dimensional convolutional layers to extract local spatial features. The second layer used twice as many filters to enhance feature representation. A residual connection was added by mapping the input features to the same dimension and combining them with the output through element-wise addition. This helps retain low-level information and improves the fusion of shallow and deep features, enhancing model performance.

**Figure 6:**
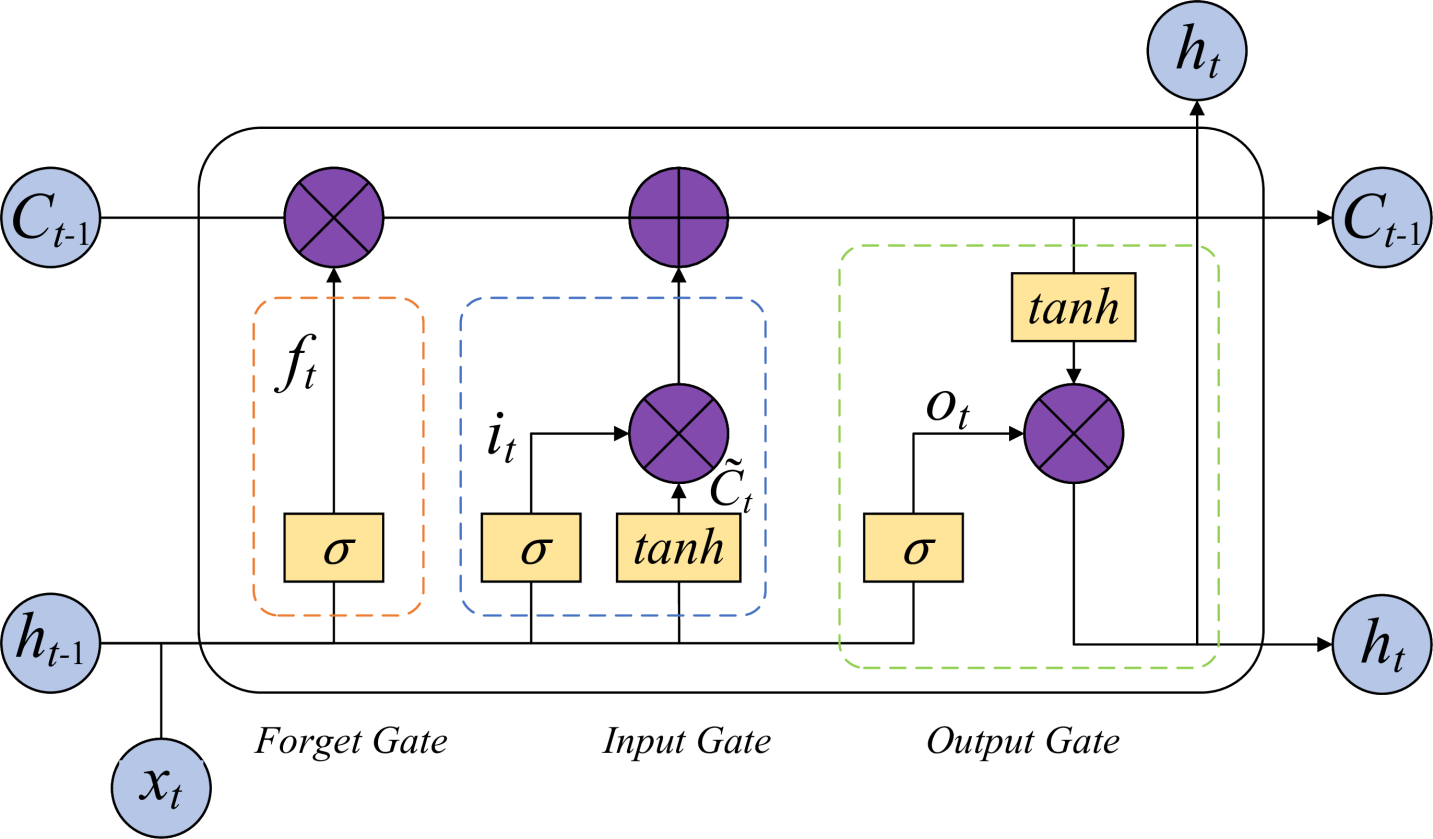
The internal chain structure of an LSTM unit.

After extracting spatial features with CNN, the sequential representation of crown height layers was passed into an LSTM layer to model vertical structural variation. The LSTM processes normalized input features as ordered steps corresponding to different crown heights. By maintaining and updating hidden and cell states across time steps (**Figure 6**), the LSTM captures the dependency between consecutive height layers, enabling the model to learn how crown radius evolves from the lower canopy to the upper layers. This sequential processing allows the network to retain structural patterns over the entire crown profile, thereby improving the prediction accuracy of crown radius at each height level and enhancing its ability to reflect both gradual transitions and local variations along the vertical crown axis.

**Figure 7:**
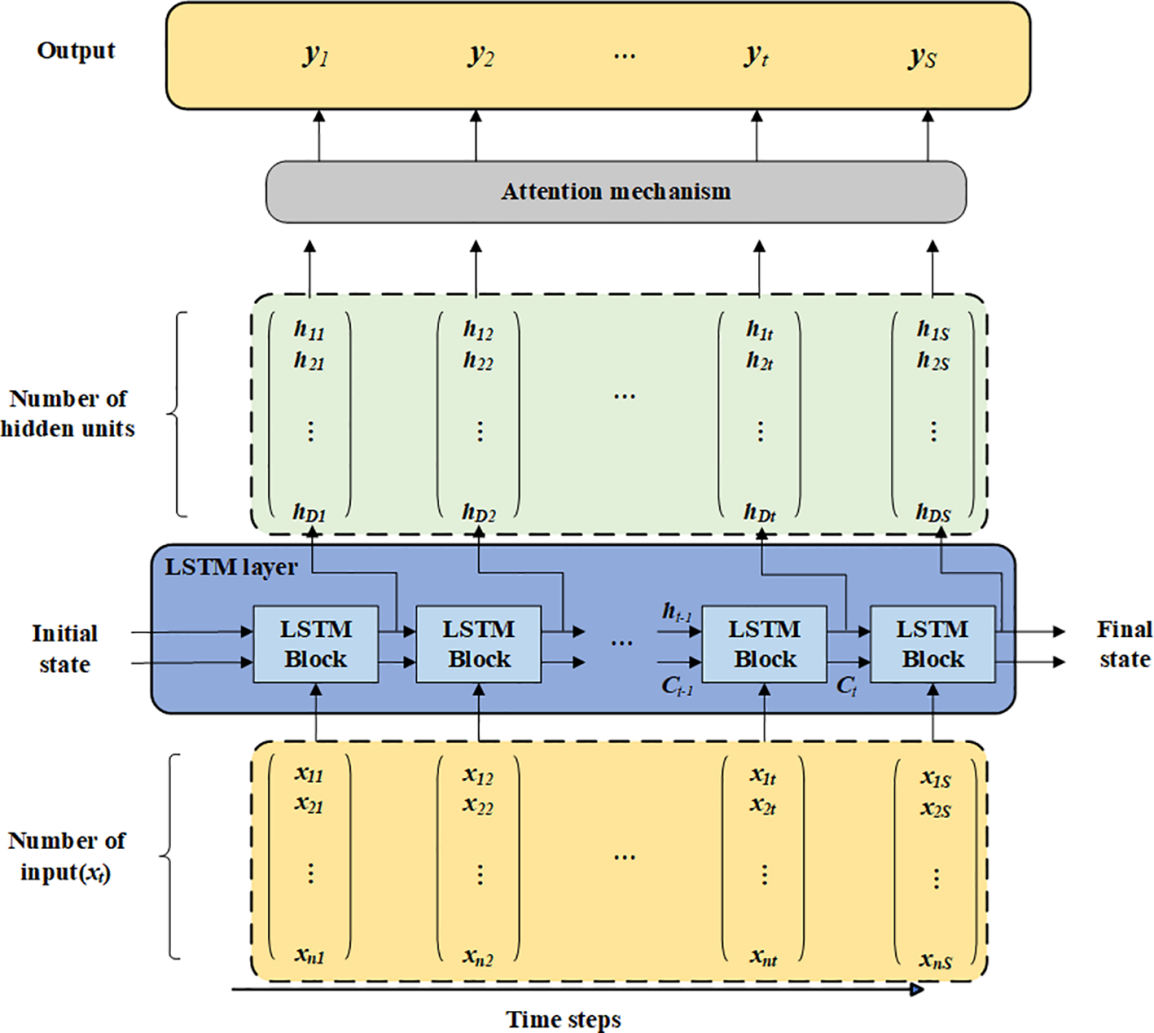
LSTM model with attention mechanism.

To further enhance the model’s ability to identify and capture key height layers within the crown profile, a time-step-based attention mechanism is introduced into the LSTM module. Traditional LSTM models typically assign equal importance to all time steps (height layers), which limits their capacity to emphasize the positions most representative for prediction. The attention mechanism addresses this by learning the relative contribution of each height layer and dynamically allocating weights, thereby guiding the model to focus on structurally informative levels. As shown in **Figure 7**, in the proposed framework, the LSTM first outputs the hidden states corresponding to each height layer. The attention mechanism then aggregates these hidden states through weighted fusion. Specifically, the hidden states are first permuted along the temporal dimension, followed by a fully connected layer and a softmax activation to generate an attention distribution over time steps. This distribution is then used to compute a weighted sum with the original LSTM outputs, producing an enhanced sequence representation. This enriched output is passed to the final output layer to predict crown radius at each height level. By enabling the model to adaptively emphasize the most informative vertical layers, the attention mechanism contributes to a more nuanced representation of crown profile, ultimately enhancing both prediction accuracy and model interpretability.

**Figure 8:**
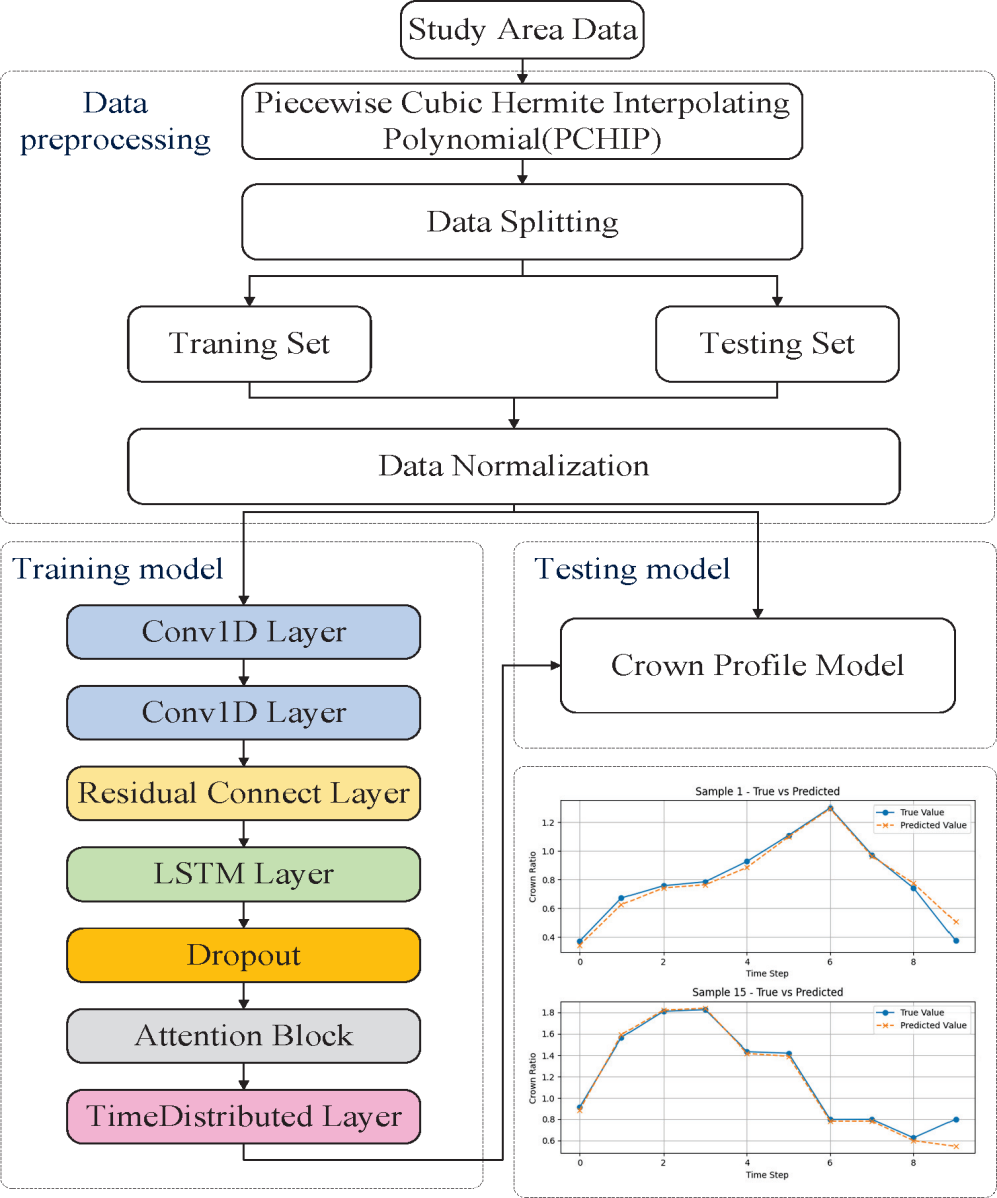
Overall experimental flow diagram.

In summary, the CNN-LSTM-Attention model integrates spatial feature extraction, sequential modeling, and adaptive attention weighting to predict crown radius across different height levels. Normalized input features are first processed through convolutional and residual layers to capture spatial characteristics. These processed features are then fed into an LSTM layer, which models the dependencies among crown height layers. The attention mechanism subsequently refines the output by emphasizing the most informative positions within the vertical crown profile. Final predictions are obtained via a Timedistributed layer and reverse normalization. The overall modeling process is illustrated in **Figure 8**.

Particle Swarm Optimization(PSO) is an intelligent parameter optimization algorithm proposed by Eberhart and Kennedy in 1995 (Kennedy and Eberhart, 1995). It is inspired by the swarm behavior of birds and fish. It simulates the cooperation and competition between individuals (particles) in the search space to find the optimal solution to the problem. The basic process of PSO includes initialization, iteration update, location update, evaluation and selection, and termination condition judgment.

All models finally constructed in this study are optimized by pyswarm library’s particle swarm optimization algorithm for hyperparameters. The ranges of hyperparameters were determined based on preliminary tests and computational efficiency considerations, and were set as follows: filters: 32 to 256; LSTM units: 32 to 128; learning rate: 1e-5 to 1e-2; Batch size: 16 to 64.

### 2.5 Model evaluation and validation

The data set in this study adopts a segmentation ratio of 8:1:1, that is, 80% of the original data is used for training model parameters, 10% of the data is used as a validation set for optimization during model training, and 10% of the test set is used to evaluate the predictive performance of the model. All models were trained and tested on computers running the Windows 11 operating system, equipped with the GPU RTX 4060 and 32GB of memory. Model performance was evaluated by determining the values of coefficient (R², **Equation 8**), root mean square error (RMSE, **Equation 9**), mean square error (MSE, **Equation 10**), mean absolute deviation (MAE, **Equation 11**), and mean error (ME, **Equation 12**).


(8) $$\begin{array}{c} R^{2}=1-\frac{\sum_{i=1}^{n}{\left(y_{i}-{\hat{y}}_{i}\right)}^{2}}{\sum_{i=1}^{n}{\left(y_{i}-{\bar{y}}_{i}\right)}^{2}} \end{array}$$

(9) $$\begin{array}{c} RMSE=\sqrt{\frac{\sum_{i=1}^{n}{\left(y_{i}-{\hat{y}}_{i}\right)}^{2}}{n}} \end{array}$$

(10) $$\begin{array}{c} MSE=\frac{1}{n}\sum_{i=1}^{n}{\left(y_{i}-{\hat{y}}_{i}\right)}^{2} \end{array}$$

(11) $$\begin{array}{c} MAE=\frac{\sum_{i=1}^{n}\left|y_{i}-{\hat{y}}_{i}\right|}{n} \end{array}$$

(12) $$\begin{array}{c} ME=\frac{\sum_{i=0}^{n}\left(y_{i}-{\hat{y}}_{i}\right)}{n} \end{array}$$

Where $y_{i}$ represents the observed value of the i th sample; ${\hat{y}}_{i}$ is the predicted value of the i th observation; n is the number of samples and ${\bar{y}}_{i}$ is the average of all observed samples.

## 3 Results and analysis

### 3.1 Prediction accuracy of the crown profile model

The hyperparameters of all models are adjusted by PSO algorithm to ensure that the hyperparameters of each model can achieve the best performance in training and prediction. To guarantee the effectiveness of the optimization results, the same hyperparameter ranges and optimization strategies were applied throughout the optimization process. By jointly optimizing multiple hyperparameters (filters, number of LSTM units, learning rate, and batch size), we ultimately obtained a combination of hyperparameters that performed best on the validation set. These optimized parameters not only enhance the training performance of the model but also significantly improve its generalization ability. The results of all hyperparameter optimizations employed in the experiments are summarized in **Table 3**.

**Table 3:** The parameter values for different models.

Direction	Model	Filters	LSTM units	Learning rate	Batch size
No	Vanilla LSTM	—	150	0.003	22
	CNN-LSTM	130	127	0.002	45
	CNN-LSTM-Attention	148	97	0.002	40
East	Vanilla LSTM	—	146	0.003	30
	CNN-LSTM	67	63	0.002	52
	CNN-LSTM-Attention	49	83	0.007	56
West	Vanilla LSTM	—	105	0.005	33
	CNN-LSTM	79	95	0.007	19
	CNN-LSTM-Attention	172	59	0.002	34
South	Vanilla LSTM	—	105	0.005	33
	CNN-LSTM	59	121	0.003	43
	CNN-LSTM-Attention	66	114	0.001	16
North	Vanilla LSTM	—	67	0.006	47
	CNN-LSTM	148	116	0.002	47
	CNN-LSTM-Attention	143	128	0.002	23

Based on three different crown profile prediction models, **Table 4** demonstrates significant differences in their performance when CPCI is not incorporated. The performance of the Vanilla LSTM model was relatively poor, with MSE of 0.07771 m^2^, RMSE of 0.27877 m, and R² of only 0.81079. This indicates that the model was weak in fitting the crown profile and had a large error. In contrast, the CNN-LSTM model showed a better prediction effect, with MSE decreasing significantly to 0.01176 m^2^, RMSE decreasing significantly to 0.10847 m, and R² increasing to 0.97611. This suggests that it has obvious advantages in capturing crown profile features. The CNN-LSTM-Attention model performed the best without the addition of CPCI, achieving MSE of only 0.00755 m^2^, RMSE of 0.08691 m, and R² of 0.98161. This indicates that the model could more accurately fit the crown profile data with the smallest prediction error. As shown in **Table 5**, after the introduction of CPCI, the performance of the three models improved to varying degrees, indicating that CPCI plays a positive role in the process of crown radius prediction. The MSE of the Vanilla LSTM decreased from 0.07771 m^2^ to 0.05628 m^2^, and the R² increased from 0.81079 to 0.86297. This demonstrates that the model error was reduced and the fitting effect was improved after the introduction of CPCI, although the overall performance remained insufficient. The MSE of the hybrid CNN-LSTM model decreased from 0.01176 m^2^ to 0.00712 m^2^, and the R² increased from 0.97611 to 0.98266, further verifying the enhancement effect of CPCI on model accuracy. The CNN-LSTM-Attention model was further optimized with the introduction of CPCI, with the MSE dropping to 0.00618 m^2^ and the R² reaching 0.98496, demonstrating the optimal prediction accuracy and fitting effect.

**Table 4:** Comparison of test results of different crown profile model (no CPCI).

Model	MSE (m^2^)	RMSE (m)	MAE (m)	ME (m)	R^2^
Vanilla LSTM	0.07771	0.27877	0.19362	-0.00809	0.81079
CNN-LSTM	0.01176	0.10847	0.05277	**0.00234**	0.97611
CNN-LSTM-Attention	**0.00755**	**0.08691**	**0.05198**	-0.01142	**0.98161**

Best performance is highlighted in bold.

**Table 5:** Comparison of test results of different crown profile model (CPCI).

Model	MSE (m^2^)	RMSE (m)	MAE (m)	ME (m)	R^2^
Vanilla LSTM	0.05628	0.23724	0.16520	**0.00049**	0.86297
CNN-LSTM	0.00712	0.08439	0.05259	-0.02277	0.98266
CNN-LSTM-Attention	**0.00618**	**0.07861**	**0.04474**	-0.01017	**0.98496**

Best performance is highlighted in bold.

As can be seen from the results in **Table 6**, when CPCI is not added, there are obvious differences in the performance of the three models in the data sets in the four directions of east, south, west and north. The CNN-LSTM-Attention model has the best performance in all directions. Its MSE in the north is 0.02557 m^2^, and its R² is as high as 0.96926, while its MSE in the east is only 0.03902 m^2^, and its R² is as high as 0.96675. The results show that the CNN-LSTM-Attention model has strong fitting ability on data sets with different directions. The CNN-LSTM model is second, for example, the MSE of the north is 0.02593 m^2^, the R² is 0.96883, and the MSE of the east is 0.05452 m^2^, the R² is 0.95945, indicating that this model also has a good ability in directional feature extraction. The Vanilla LSTM model has a significantly weaker performance, such as a north MSE of 0.30076 m^2^ and an R² of 0.63848, and an east MSE of 0.40557 m^2^ and an R² of only 0.65446, indicating its limited ability to capture advanced features.

**Table 6:** Comparison of the performance of each crown profile model in different directions (no CPCI).

Model	Direction	MSE (m^2^)	RMSE (m)	MAE (m)	ME (m)	R^2^
Vanilla LSTM	East	0.40557	0.63684	0.37694	-0.03718	0.65446
	West	0.25251	0.50250	0.30544	-0.00583	0.67570
	South	0.40988	0.64022	0.37632	0.01959	0.66544
	North	0.30076	0.54841	0.33521	-0.04107	0.63848
CNN-LSTM	East	0.05452	0.23350	0.11332	0.04749	0.95355
	West	0.05037	0.22443	0.10594	0.00688	0.95145
	South	0.09246	0.30408	0.10966	0.00476	0.92453
	North	0.02593	0.16104	0.07523	0.02649	0.96883
CNN-LSTM-Attention	East	0.03902	0.19754	0.08169	0.00631	0.96675
	West	0.03780	0.19443	0.08217	0.04735	0.95709
	South	0.08914	0.29856	0.10789	0.00945	0.92724
	North	0.02557	0.15992	0.07415	-0.01807	0.96926

**Table 7** show the results after CPCI is added. Compared with the results without CPCI, the performance of the three models has been significantly improved, but the degree of improvement is different. The performance of the CNN-LSTM-Attention model was further optimized in all directions, for example, the southbound MSE decreased from 0.08914 m^2^ to 0.04862 m^2^, the R² increased from 0.92724 to 0.96032, and the eastbound MSE decreased to 0.04006 m^2^, the R² increased to 0.96033. The performance of CNN-LSTM model is also improved to some extent, for example, the northbound MSE is reduced to 0.02221 m^2^, the R² is increased to 0.97330, and the eastbound MSE is 0.04168 m^2^, the R² is 0.95988, indicating that its directional feature capturing ability is further enhanced. In contrast, the improvement of the Vanilla LSTM model is smaller, for example, the north MSE is reduced from 0.30076 m^2^ to 0.26741 m^2^, and the R² is only increased from 0.63848 to 0.67856, while the east MSE is reduced from 0.40557 m^2^ to 0.31187 m^2^, and the R² is increased from 0.73429. But overall performance still lags significantly behind the other two models.

**Table 7:** Comparison of the performance of each crown profile model in different directions (CPCI).

Model	Direction	MSE (m^2^)	RMSE (m)	MAE (m)	ME (m)	R^2^
Vanilla LSTM	East	0.31187	0.55845	0.34073	-0.01333	0.73429
	West	0.22502	0.47436	0.28813	-0.03212	0.71101
	South	0.34459	0.58702	0.35328	0.01873	0.71873
	North	0.26741	0.51712	0.33258	-0.04279	0.67856
CNN-LSTM	East	0.04168	0.20415	0.09487	-0.00598	0.96449
	West	0.02894	0.17012	0.08040	-0.00284	0.96283
	South	0.07090	0.26628	0.10944	0.00485	0.94213
	North	0.02221	0.14904	0.06432	-0.02296	0.97330
CNN-LSTM-Attention	East	0.04006	0.20015	0.09700	0.00124	0.96587
	West	0.02556	0.15988	0.07037	0.01577	0.96717
	South	0.04862	0.22049	0.09462	0.00319	0.96032
	North	0.02117	0.14551	0.06450	-0.00057	0.97455

In general, the CNN-LSTM-Attention model demonstrates the most stable predictive performance across all directional datasets, as indicated by multiple evaluation metrics. The CNN-LSTM model performs slightly less effectively, while the Vanilla LSTM exhibits the lowest accuracy and limited robustness to directional variation. Incorporating CPCI leads to further improvements across all models. The enhancement is most pronounced in the Vanilla LSTM, while CNN-LSTM and CNN-LSTM-Attention also benefit, confirming the effectiveness of CPCI in enhancing the stability and accuracy of crown profile predictions.

### 3.2 Analysis of the relative importance of different factors to tree crown radius

**Figure 9:**
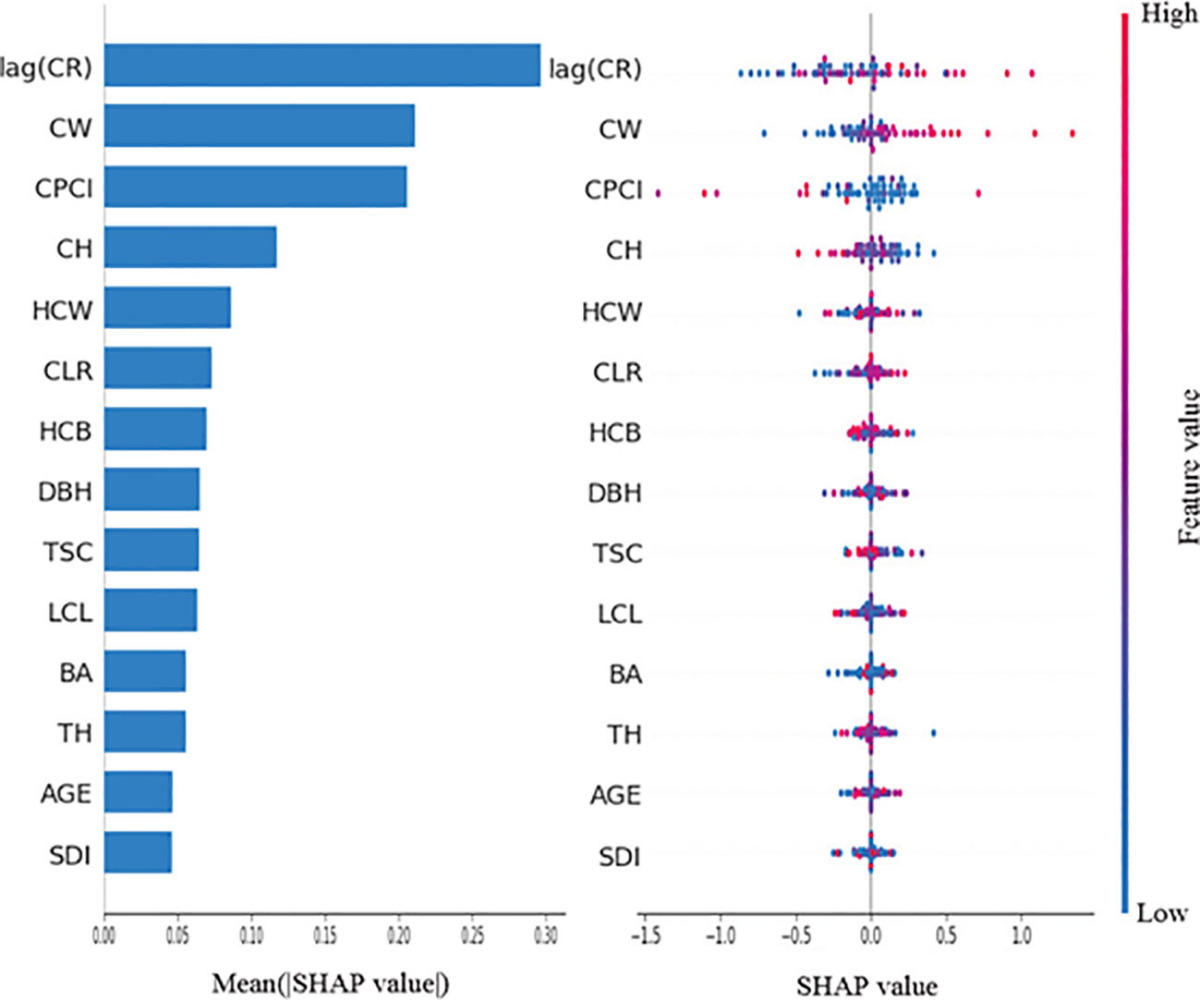
Feature importance plot and swarm plot for Vanilla LSTM model.

**Figure 10:**
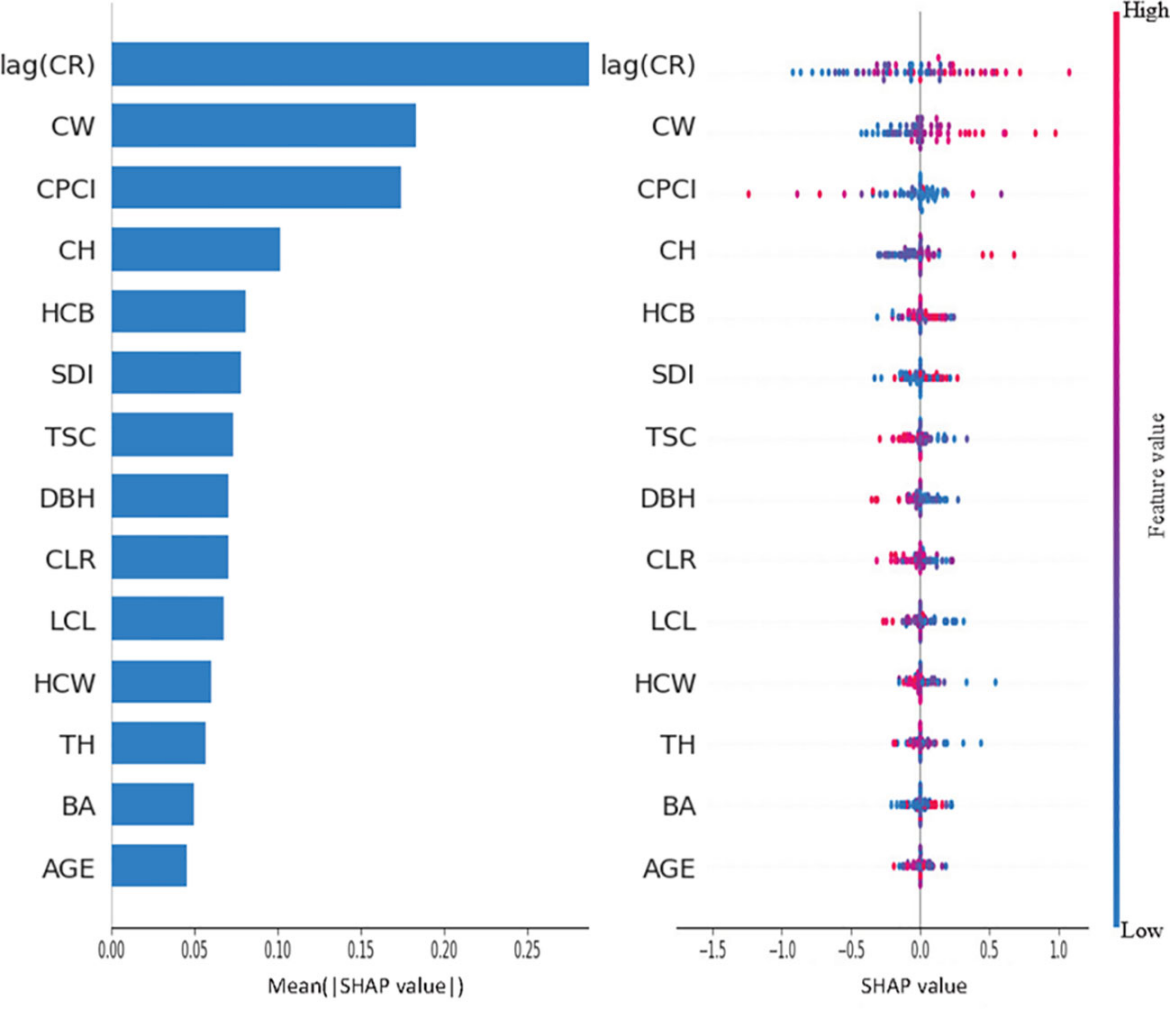
Feature importance plot and swarm plot for CNN-LSTM Attention model.

In this study, the unified framework SHAP is used to interpret the predictions of deep learning models. As can be seen from **Figures 9**, **10**, there are obvious differences between Vanilla LSTM and CNN-LSTM-Attention models in the importance of features and their effects on predictor variables. For Vanilla LSTM models, lag(CR) is the most important feature and has a significant positive correlation with the predictor variables. CW ranked second, and its larger value also showed a strong positive correlation with the predictor variables, indicating that the larger the crown width, the higher the predicted value of the current crown radius. CPCI ranks third, and its larger value has a significant negative correlation with the predictor. CH also showed a negative correlation to the predictor, while HCW had a weak effect, but still showed a positive correlation trend. Other features such as CLR, HCB, DBH, etc. have low correlation to the predictor variables, and their contribution is relatively limited.

By contrast, the CNN-LSTM-Attention model makes more comprehensive use of features. lag(CR) is still the most important feature and has a significant positive correlation with the predictor variables. CW and CPCI followed closely behind, with CW showing a strong positive correlation to the predictor, while CPCI had a strong negative correlation. Compared with Vanilla LSTM model, the importance of HCB and SDI in CNN-LSTM-Attention model has increased significantly, where HCB has a negative correlation with the predictor variables, while SDI has a strong positive correlation. The distribution of SHAP values for other variables such as CLR, TSC, LCL, HCW, and TH shows that most of the blue dot values are concentrated in the region where SHAP is greater than 0, indicating that these variables have a certain negative correlation with the predictor in general. The feature importance of AGE and BA is at a low level in both models, indicating that these two variables have little impact on the prediction results. However, from the distribution of SHAP values in the two graphs, it can be seen that in Vanilla LSTM and CNN-LSTM-Attention models, some small values of AGE and BA correspond to regions with SHAP values less than 0, indicating that when their values are small, the predicted value of crown radius will be reduced.

## 4 Discussion

In forest investigation, crown profile database or trunk morphology database belongs to the category of hierarchical data, which is similar to time series data and can be regarded as time series data. Under this data structure, although the Vanilla LSTM model can handle the temporal dependence in time series, its processing ability for hierarchical data structures is limited. Moreover, the Vanilla LSTM model can only rely on the original time series features and cannot effectively consider the influences between different levels. Although the Vanilla LSTM model can obtain better results in some simple tasks, it has lower prediction accuracy when it is faced with tree crown profile prediction which contains complex hierarchical relationships and multi-dimensional data. Therefore, this study proposed a hybrid deep learning method based on the attention mechanism of CNN and LSTM to model the crown profile of *Pinus yunnanensis*. The proposed model can extract advanced features efficiently, fuse original features with advanced features, and improve its prediction performance. From the perspective of result analysis, the CNN-LSTM-Attention model proposed can indeed integrate the competition between different levels well, and its adaptability to data hierarchical structure is stronger than the Vanilla LSTM model, no matter the data sets with no direction or data sets with all directions.

The Traditional CI is widely used in forestry to describe the competitive relationships between trees, typically calculating competition intensity based on the distance between trees and biological characteristics such as tree height and diameter at breast height (Firmino et al., 2023; Wang et al., 2024). While this method is effective in simple environments, its shortcomings become increasingly apparent in complex ecosystems. Firstly, the traditional CI treats competition as a holistic phenomenon, neglecting spatial and directional heterogeneity (Hui et al., 2018). For example, different directions of a tree’s crown may exhibit varying growth patterns and competitive effects, resulting in less precise predictions in complex environments. Secondly, the calculation methods of traditional CI are relatively simplistic, considering only horizontal spatial relationships and ignoring vertical competition (Wang et al., 2015; Kobal and Levanič, 2025). Shorter trees may be affected by shading from taller trees, while taller trees may face limitations in light availability and spatial occupancy. These factors are difficult to fully capture within traditional CI. Therefore, the traditional CI has certain limitations in crown profile modeling and the management of complex competitive relationships.

Due to the hierarchical data structure of the crown profile dataset in this study, and in order to better describe the competitive effects of trees at different height levels, the proposed CPCI calculates the height differences between trees and adjusts the calculation of crown overlap areas at each level accordingly. This approach allows for the consideration of spatial distribution while accurately reflecting competitive conditions at various height levels. In this way, it not only accounts for the spatial overlap of tree crowns but also incorporates the influence of tree height on competition, thereby enabling a more precise description of competitive relationships between different trees. Incorporating CPCI into various models results in performance improvements to varying degrees across all models.

Additionally, factors such as terrain undulation, wind direction, and the distribution of neighboring trees result in varying intensities and durations of sunlight exposure for trees in the east, south, west, and north directions. This causes crowns to be denser and more expansive in directions with ample sunlight, while crowns in shaded directions remain relatively sparse. Scatter plots (**Figure 3**) reveal that the crown profiles of *Pinus yunnanensis* exhibit directional asymmetry, with crown radii in the southern direction being larger than those in other directions. This pattern may stem from the predominant low solar angles favoring southern exposure in the Northern Hemisphere, where trees in higher latitudes often develop taller branches and needle structures on their southern crowns (Eklund and Säll, 2000; Rouvinen and Kuuluvainen, 1997). Conversely, trees growing freely in lower latitudes are expected to form more symmetrical crowns (Brisson, 2001). Traditional crown profile models typically assume crown symmetry, overlooking these directional differences, which often leads to less accurate predictions of crown contours. Therefore, constructing crown profile models tailored to different directions and developing competition indices that describe or quantify competitive pressures from various orientations is particularly necessary. Comparative performance analysis of crown profile models across different directions and the relative importance of factors indicate that, after allocating competitive pressures in various directions, the adjusted competition index factors retain high importance for the prediction variables in the models and significantly enhance model performance.

Future studies can further improve the robustness and accuracy of the model by refining the model structure and optimizing the calculation method of the crown profile competition index, particularly the directional allocation scheme, to more accurately capture the variation in crown profile across different directions. However, the current analysis is based solely on data from permanent sample plots located on Cangshan Mountain in Dali, Yunnan Province, which imposes geographic and species-specific limitations. Crown development is influenced by a variety of factors such as site conditions, interspecific competition, and silvicultural practices, which can vary significantly across ecological regions. Due to the limited availability of tree-level data from other areas and species, it is not yet feasible to incorporate such information into the current model. Nevertheless, when such data become available, future research will aim to validate the model across broader ecological contexts to assess its transferability and improve its generalizability and applicability to diverse forest management scenarios.

## 5 Conclusion

In this study, we developed a deep learning model that hybrids CNN with LSTM for the task of crown profile prediction. Building upon this foundation, an attention mechanism is further incorporated to enhance the model’s focus on key features. Comparative analysis with the Vanilla LSTM model reveals that the CNN-LSTM model not only more effectively extracts spatial structural features but also achieves improved performance. Furthermore, the addition of the attention mechanism to the CNN-LSTM model results in the CNN-LSTM-Attention model, which further enhances prediction accuracy and model stability. Particularly, in prediction tasks involving crown profile datasets from different directions, the CNN-LSTM-Attention model demonstrates the best results, fully illustrating its applicability and robustness in complex data scenarios. Additionally, we propose a CPCI tailored for crown profiles. The introduction of CPCI effectively enhances the performance of the Vanilla LSTM, CNN-LSTM, and CNN-LSTM-Attention models. In summary, this paper makes the following contributions:

1. Constructed effective crown profile models for both non-directional and directional crown profile databases of *Pinus yunnanensis*.
2. The proposed hybrid CNN-LSTM model based on the self-attention mechanism achieved the best performance in the crown profile prediction task regardless of direction and in different directions.
3. A crown profile-oriented competition index is proposed, and its performance is improved when introduced into multiple models.

## Data Availability

The raw data supporting the conclusions of this article will be made available by the authors, without undue reservation.
